# A Comparative Analysis of Biodegradation and Bioconversion of *Lentinula edodes* and Other Exotic Mushrooms

**DOI:** 10.3390/microorganisms11040897

**Published:** 2023-03-30

**Authors:** Diego Cunha Zied, Marcos Antônio da Silva Freitas, Bruno Rafael de Almeida Moreira, Lucas da Silva Alves, Arturo Pardo-Giménez

**Affiliations:** 1 Department of Plant Production, College of Agricultural and Technological Sciences, São Paulo State University (Unesp), Dracena 17900-000, SP, Brazil; 2 Department of Engineering and Exact Sciences, School of Agricultural and Veterinary Sciences, São Paulo State University (Unesp), Jaboticabal 14884-900, SP, Brazil; 3 Department of Applied Microbiology, School of Agricultural and Veterinary Sciences, São Paulo State University (Unesp), Jaboticabal 14884-900, SP, Brazil; 4 Centro de Investigación, Experimentación y Servicios del Champiñón (CIES), 16220 Quintanar del Rey, Spain

**Keywords:** *Agrocybe aegerita*, *Flammulina velutipes*, *Pleurotus eryngii*, shiitake

## Abstract

Mushrooms are capable of bioconverting organic residues into food. Understanding the relationship between high-quality yields and substrate biomass from these residues is critical for mushroom farms when choosing new strains. The objective of this exploratory study was, therefore, to analyze whether exotic mushrooms, namely, *Pleurotus eryngii*, *Flammulina velutipes*, and *Agrocybe aegerita*, could biologically convert the substrate into edible mushrooms as effectively as *Lentinula edodes* (baseline). Five experiments were carried out. Biological efficiency, biodegradability coefficient, mass balance and chemical characterization of the substrate were evaluated. Strategically hydrating the sawdust enabled *L. edodes* to achieve the greatest biodegradability and biological efficiency of 0.5 and 94.2 kg dt^−1^, respectively. The values for *L. edodes* on wheat straw without hydration were 0.2 and 68.8 kg dt^−1^, respectively. From 1000 kg of fresh substrate, *P. eryngii* produced 150.1 kg of edible mushrooms, making it technically competitive with *L. edodes* on wheat straw (195.9 kg). Hence, *P. eryngii* was the most reliable option for scaling among the exotic mushrooms. The analytical insights from our study provide further knowledge to advance the field’s prominence in high-throughput mushroom-producing systems, particularly for exotic mushrooms.

## 1. Introduction

The Food and Agriculture Organization’s experts estimate that the world’s population will be 10 billion people by 2050, and the agriculture and food security ecosystem must be aware of the commitment, cooperation, and coordination necessary to effectively feed people sustainably in the future [1]. Therefore, for every stakeholder [2], how to feed people sustainably is humanity’s most fundamental challenge and will likely become harder to solve in the near future with an increasing population and changing global climate.

Agricultural crops are influenced daily by abiotic and biotic factors [3]. One way to reduce these impacts is to grow crops in protected environments but this is not always possible. Crops that require large expanses of land or acreage of land, such as soybeans, wheat and corn, are examples of this [4,5]. However other crops, such as horticultural crops, and mushrooms are amenable to cultivation in protected environments. Mushrooms are nutritious foods that provide vitamin D, minerals, dietary fiber, and protein and can contribute to fulfilling the requirements for food and nutrient security of Agenda 2030 for Sustainable Development [6,7]. Some critical components found in mushrooms are minerals and antioxidants that help to prevent hidden hunger, and some components are medicinal.

The global edible mushroom industry is structurally heterogeneous. It consists of an extensive range of actors, kinds of production, and agribusiness models, from smallholder farming to high-tech production systems [8,9]. The most commercially valuable genera of mushroom-producing fungi worldwide are *Agaricus* spp. [7,10,11], *Lentinula* spp. [12], and *Pleurotus* spp. [7]. 

Shiitake (*L. edodes*) ranks first in the world’s production of edible mushrooms [13]. Its mycelium can secrete lignin-degrading enzymes [14], making it capable of producing fruiting bodies on wood or any similar artificial material that is cost effective [15]. Thus, the traditional cultivation of shiitake on oak logs has been replaced in part by cultivation in plastic bags with sterilized enriched sawdust since this method offers a higher biological efficiency and a shorter cultivation cycle. Moreover, the feasibility of cultivating shiitake in pasteurized straw offers a very important alternative since straw is generally an easily accessible substrate [16].

Water availability during shiitake cultivation is probably the most important factor influencing growth and fruiting body production [17]. At the end of the harvest of each flush, blocks can be soaked in water to rehydrate the substrate and induce the development of fruiting bodies for the next flush [18]. This method is frequently used since mycelial growth compacts the substrate and hinders hydration of the samples by other means [16]. 

*Pleurotus eryngii*, *Flammulina velutipes*, and *Agrocybe aegerita* are exotic species cultivated in Asia [7,19]. Similar to *L. edodes*, they produce of lignin-degrading enzymes. Hence, they can grow on wood trunks and straw in nature. Most notably, they can adapt to a wide range of commercial substrates or even agricultural, agro-industrial, or forestry waste, recycling them into protein-rich biomass via biodegradation and biotransformation. 

Tea waste [20], millet, rice, wheat, barley, oat straw [21,22], spent coffee grounds [23], fruit peels [24], and food waste digestate [25,26] can serve as a raw base for cultivation material. Each kind of material used will have an impact on production parameters, such as mycelium run period, method of sterilization, total cultivation time, productivity, presence of contaminants, and amount of mushroom waste.

The efficiency of production is related to reducing the generation of waste at the end of the crop time and improving the production environment to achieve the highest and best mushroom quality for a given substrate mass [21]. Intensive production of edible mushrooms is often conducive to the occurrence of diseases and pests in fungus-growing waste material, even if it is sterile and consists of a sufficient quantity of nutrients for mycelial growth [27]. Thus, the level of waste generation and the quantity of mushrooms produced must be carefully characterized. 

The primary objective of our study was to analyze whether exotic mushrooms, namely, *P. eryngii*, *F. velutipes*, and *A. aegerita*, could biologically convert lignocellulosic substrate to edible mushrooms as effectively as the reference species, *L. edodes*. The secondary objective was to compare the bioconversion of lignocellulose and other components of the substrates by *L. edodes* in a growing system on pasteurized wheat straw-based substrates without hydration.

## 2. Materials and Methods

### 2.1. Experimental Design

Four cultivated species of edible mushrooms were studied: *L. edodes* (Berk.) Pegler, *A. aegerita* (V. Brig.) Vizzini, *F. velutipes* (Curtis) Singer, and *P. eryngii* (DC.) Quél. *Lentinula edodes* was cultivated under two different growing systems: a sterilized substrate with additional hydration, and a pasteurized substrate without hydration. Thus, five independent growing cycles were carried out in experimental climate-controlled growing rooms. Initially, the chemical characterization of the substrates in each experiment (Table S1, Supplementary Material) was carried out in duplicate according to the methodology described below. In each experiment, six bags of substrate were selected, which were weighed at the beginning of the cycle, monitored for their productivity (biological efficiency), and weighed again. Finally, the spent mushroom substrate of each bag was chemically characterized.

Biological efficiency (BE, kg dt^−1^), a practical estimate of the ability of mushrooms to convert substrate into fruiting bodies, was calculated according to Equation (1) [28]:

BE = (TWFM/DWS)⋅100(1)

where TWFM is the total weight of fresh mushrooms harvested (kg), and DWS is the dry weight of substrate (kg). The biodegradability coefficient (Km) was defined according to Equation (2) [29]:

Km = OML/OMI(2)

where OML is the organic matter lost from the process (kg), and OMI is the organic matter input into the process (kg). Mass balances were calculated based on the initial and final weights of fresh substrates before and after the growing cycles and their analytical composition was determined. The productivity of the different cultivated species, and the composition of fruiting bodies were also assessed. 

### 2.2. Individual Growing Cycle Description

The spawn used in all the experiments described below was prepared using wheat grain supplemented with 2% CaCO_3_ for the different strains. The inoculation rate was 2% (*w*/*w*) for all experiments.

In the first experiment testing *L. edodes*, the Fungisem S-5-3 strain (Fungisem Micelios S.A., Autol, Spain) was inoculated on a sterilized sawdust-based commercial substrate. The substrate was deposited in plastic bags with a 3.3 kg wet weight and incubated prior to filling in the growing room. The substrates were then matured for 33 days (T 24 ± 1 °C; RH 85–90%; CO_2_ > 2000 ppm), after which they were immersed in water (24 h, 15 °C), followed by fruiting induction (T 15–16 °C; RH 90%; CO_2_ 800–1000 ppm; light 200–500 lux). The mean weight of the bags after hydration was 5.4 kg bag^−1^. Before hydration, the plastic bag was removed, and harvest began 43 days after filling. Two additional hydrations of the substrate were performed between flushes. The total duration of the growth cycle was 95 days.

In the second experiment testing *L. edodes*, the same strain from the previous experiment, Fungisem S-5-3, was used to inoculate a pasteurized wheat straw-based commercial substrate. The substrate was deposited in plastic bags with a 17.4 kg wet weight and incubated prior to filling in the growing room. After filling, fruiting induction conditions were directly applied (T 16–18 °C; RH 85–90%; CO_2_ 1000–1200 ppm, light 200–500 lux). To induce flushing, the plastic bag was removed and harvest began 7 days later. The total length of the growing cycle was 62 days.

In the third experiment testing *P. eryngii*, the Fungisem ER-24 strain (Fungisem Micelios S.A., Autol, Spain) was used to inoculate a sterilized sawdust-based commercial substrate. The substrate was deposited in plastic bags with a 3.1 kg wet weight and incubated prior to filling in the growing room. After filling, the room was kept under vegetative growth conditions for four days (T 22–23 °C; RH 90–95%; CO_2_ > 2000 ppm), after which fruiting induction was carried out (T 16–17 °C; RH 85–90%; CO_2_ 700–800 ppm; light 200–500 lux). To induce flushing the plastic bag was cut superficially. Harvest began 13 days after filling, and the total length of the growing cycle was 38 days. 

In the fourth experiment (*F. velutipes*), the Gurelan FV strain (Gurelan Mycelium S.C., Huarte, Spain) was used to inoculate a sterilized sawdust-based commercial substrate. The substrate was deposited in plastic bags with a 4.1 kg wet weight and incubated prior to filling in the growing room. After filling, fruiting induction conditions were directly applied (T 14–15 °C; RH 85–90%; CO_2_ 1000–1200 ppm, light 200–500 lux). To induce flushing, the plastic bag was cut superficially. Harvest began 17 days after filling, and the total length of the growing cycle was 43 days.

In the fifth experiment testing *A. aegerita*, the Gurelan AA9 strain (Gurelan Mycelium S.C., Huarte, Spain) was used to inoculate a sterilized sawdust-based commercial substrate. The substrate was deposited in plastic bags with a 4.1 kg wet weight and incubated prior to filling in the growing room. After filling, the room was kept under vegetative growth conditions for three days (T 22–23 °C; RH 90–95%; CO_2_ > 2000 ppm), after which fruiting induction was carried out (T 15–16 °C; RH 85–90%; CO_2_ 600–700 ppm; light 100–250 lux). To induce flushing, the plastic bag was cut superficially. Harvest began 13 days after filling, and the total length of the growing cycle was 51 days. 

### 2.3. Chemical Analysis

To determine the chemical characteristics of the substrates, the following measurements were taken: pH; moisture content; organic matter and ash; total N content and crude protein; C/N ratio; crude fiber; crude fat; N-free extracts; total carbohydrates; acid detergent fiber; neutral detergent fiber; cellulose, hemicellulose; lignin, and neutral detergent soluble, following the methodology proposed by Zied et al. [30]. For the fruiting bodies, the water content, dry matter, organic matter, and ash were also determined [31,32].

### 2.4. Data Analyses

The results are presented as diagrams to allow easy comparison of the fungi by biodegradability and biological efficiency (bar plot), balance of mass (Sankey or flowchart), and substrate composition at the beginning and the end of production (arrow plot). Furthermore, contour plotting was performed to model biodegradability upon proximal, structural, and chemical properties changing with fungal cultivation, thus improving the illustration and conveying major findings and novel results of our study. The data were analyzed in the environment of the R-project for statistical computing and graphics.

## 3. Results and Discussion

### 3.1. Biodegradability and Biological Efficiency

**Figure 1:**
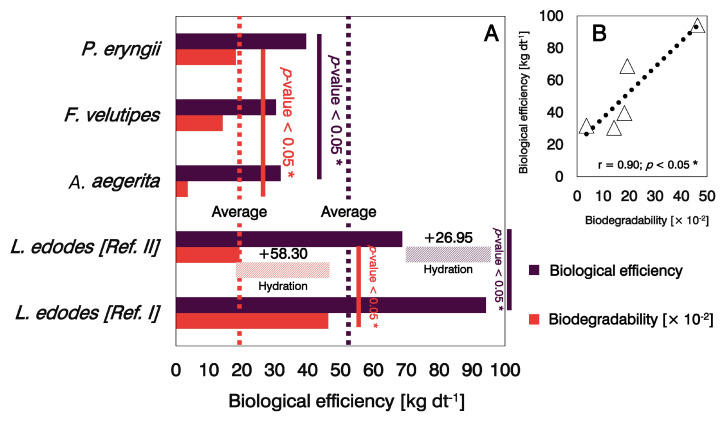
Biodegradability and biological efficiency of exotic mushrooms relative to *L. edodes* with (Ref. I) and without (Ref. II) hydration as a supplementary level of management (**A**). The functional relationship between the ability of mushrooms (*L. edodes*, *A. aegerita*, *F. velutipes*, and *P. eryngii*) to transform matter into health-promoting, nutrient-dense biomass (**B**). The larger the bar, either of biodegradability or biological efficiency, the greater the ability of the fungus to transform lignocellulosic substrate into a mass of edible mushrooms. Significance: * *p*-value < 0.05.

By analyzing the obtained data on biodegradability and biological efficiency (Figure 1), it can be observed that *L. edodes* with and without hydration effectively transformed both woody (sawdust) and non-woody (wheat straw) substrates into harvestable masses of mushrooms at 0.5 and 0.2 biodegradability, respectively (Figure 1A). Specifically, strategically introducing water into the system enabled the primary decomposer mushroom to degrade organic matter with a higher biological efficiency (94.2 kg dt^−1^) than possible when not hydrated (68.7 kg dt^−1^) through the mushroom-growing material. The physical environment for mycelial growth and fruiting varies between substrates. In the case of straw-based substrates, their structure reduces the compaction effect during mycelial growth compared with substrates based on sawdust, which facilitates its hydration by conventional irrigation. In any case, immersion hydration increased the biodegradability of *L. edodes* by 58.3% relative to that obtained with wheat straw substrate and conventional irrigation. This increase was not only influenced by hydration but also by the substrate composition. 

Comparatively, *P. eryngii* (Km = 0.2; BE = 39.6 kg dt^−1^) converted more substrate into biomass, indicating that this species was the most effective exotic mushroom for biotransformation, followed by *F. velutipes* (Km = 0.15; BE = 30.4 kg dt^−1^) and *A. aegerita* (Km = 0.05; BE = 31.8 kg dt^−1^), based on biodegradability. A study by Fornito et al. [33] on the degradative ability of mushrooms on silage consisting of corn reported incomplete colonization of the substrate by *A. aegerita*. The mycelial network of the exotic fungus was not capable of completely colonizing the substrate; thus, the bioconversion of labile C (an unstable fraction of the total C pool in raw materials) into biomass decreased, as did the biological efficiency, which is consistent with the lowest biodegradability of *A. aegerita* in this study. *P. eryngii* achieved higher levels of biotransformation compared with both *F. velutipes* and *A. aegerita*, making it technically comparable with *L. edodes* (Ref. II) in biodegradability. An effective decomposer is likely to convert more organic matter into mushrooms. However, the ability of fungi to biodegrade matter in our study could be predicted from the significant positive correlation of 0.9 between biodegradability and biological efficiency (Figure 1B). Some statistics not predicted the correlational model could be attributable to the impact of either the substrate’s qualities (e.g., nutritional composition and raw materials used) or genetic aspects of the strains (e.g., hybrid or not and whether it was adapted to the substrate).

### 3.2. Sensitivity of the Substrate to Biotransformation

**Figure 2:**
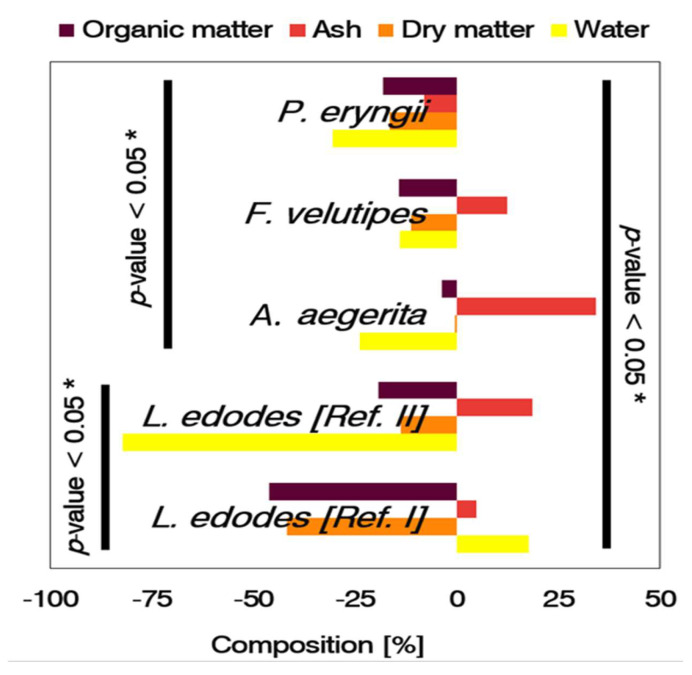
Proximal analysis of the substrate by exotic mushrooms relative to *L. edodes* with (Ref. I) and without (Ref. II) hydration as a supplementary level of management. The bars show the fluctuation in the composition of the substrate between the beginning and end of cultivation. The larger the bar, the greater the compositional distinctiveness of the spent mushroom substrate relative to the precursor material. Significance: * *p*-value < 0.05.

Mushrooms degrade the substrate matter and export part of the energy they require for fruiting into a harvestable mass. With the support of microbial metabolism, they deplete the substrate (Figure 2). By analyzing the composition of the substrate at the beginning and the end of the cropping cycle, we could characterize the fungal biotransformation and how the fungi influenced the biological process. The mushrooms biotransformed the matter in distinctive ways, and the system shaped the performance both positively and negatively. Shiitake (Ref. I) changed the proximal properties of the substrate, primarily by decreasing organic matter (−46.2%), and by relative decreases in dry matter (−41.9%) and increases in ash (4.7%) in the spent mushroom substrate, indicating mineralization.

**Figure 3:**
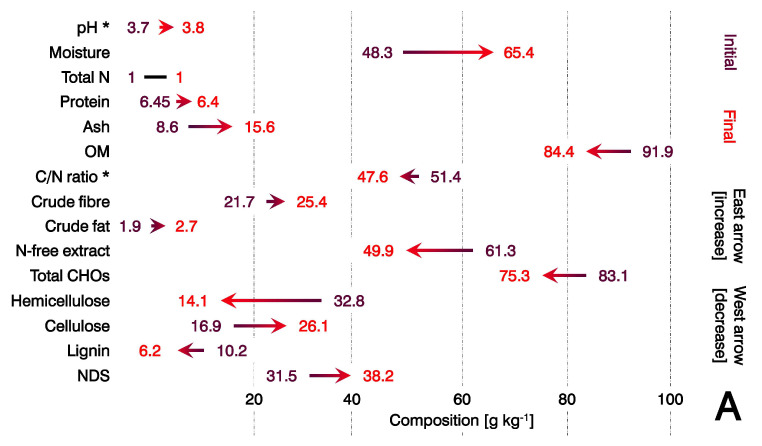

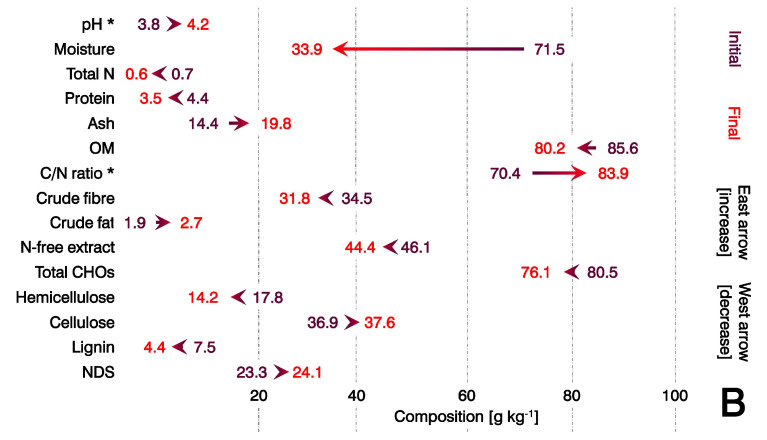
Composition of substrate at the beginning and end of systematic production of *L. edodes* in sawdust with hydration (**A**) and wheat straw without hydration (**B**). * Dimensionless variable.

The sawdust substrate (Figure 3A) consisted of 48.3 g kg^−1^ moisture, while the wheat straw substrate (Figure 3B) consisted of 71.5 g kg^−1^ moisture. However, the cereal base was likely denser in oxidisable organics (14.4 g kg^−1^ ash), enabling *L. edodes* to perform extensive mineralization and increase the ash content in the residual wheat straw by 27.3%. Although the wheat straw had greater mineralization than sawdust (Figure 2), it was not nutritionally sufficient to support competitive production relative to sawdust with hydration. However, it included larger amounts of cellulose (Figure 3B) and could provide carbohydrates as sources of metabolisable energy [34] to support the appreciable production of shiitake. Studies on producing shiitake on cereal by-products are preliminary, yet they provide knowledge to progress the formulation of non-wood substrates.

For instance, Yu et al. [35] studied the production of *L. edodes* in corncobs, stressing the importance of using an alternative substrate to wood. Because the demand for woody raw material is likely to exceed the supply in the coming years, the authors analyzed whether the corncob could be useful as a cereal base in composite substrates to produce shiitake. Substrates consisting of 50% corncob, 20% oak sawdust, 28% wheat bran, and 2% gypsum resulted in the fastest mycelial growth of the fungus, the most appealing browning on the log, and consequently, the largest production of fruiting bodies (722.1 g log^−1^) and highest biological efficiency (80.2%).

**Figure 4:**
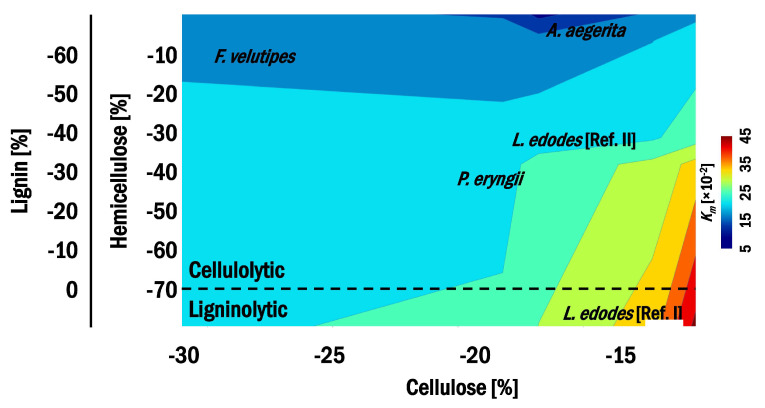
Mapping *L. edodes* and mushroom-producing exotic fungi by biodegradability (Km) on structural properties.

The structural composition, regardless of the polymer, positively affected biodegradability. Cellulose and hemicellulose were more likely to contribute to enzymatic activity than lignin, proving a predominantly cellulolytic biotransformation by *F. velutipes* and *A. aegerita*, while both *L. edodes* and *P. eryngii* performed lignocellulolytic decomposition (Figure 4).

The comparative analysis of biodegradation of lignocellulose and other components of substrates and the consequent bioconversion into fruiting bodies of different species of edible fungi focuses on advancing knowledge for several practical applications: (i) multipurpose use of substrates with different species to simplify the work and reduce costs in the substrate production facilities and raw material transport; (ii) recycling of spent mushroom substrates into new substrates for either the same or different mushroom species to allow a better use of the biomass; and (iii) optimization of substrate formulations by using chemically complementary materials to maximize biodegradation and bioefficiency in the production of fruiting bodies.

Edible mushrooms consist of a plethora of hydrolytic and ligninolytic enzymes, making them capable of hydroxylation [19]. Enzymology determines both biodegradability and biological efficiency when producing edible mushrooms on lignocellulosic substrates [36]. For instance, the mycelium of shiitake can secrete lignin-degrading enzymes, making it capable of producing fruiting bodies on wood or any similar artificial material that is cost effective [15]. 

The fungal biodegradation of lignin is an oxidative process [37], and phenol oxidases, such as peroxidases in *P. ostreatus* or any similar white-rot fungus, are key enzymes [38,39,40]. While lignin peroxidases cleave non-phenolic units in lignin, manganese peroxidases can generate Mn3+ as a diffusible oxidizer on phenolic and non-phenolic units in the polymer, primarily via peroxidation [41,42]. Copper-oxidizing laccases can catalyze biodegradation over phenolic and electron-rich substrates [43]. 

*P. eryngii* degraded more hemicellulose (−37.50%) and lignin (−23.1%) than cellulose (−18.2%). In contrast, *F. velutipes* and *A. aegerita* degraded the cellulosic fraction of the substrate, decreasing it by 31% and 16.8%, respectively, in the structural composition of the residual material. Furthermore, neither species decomposed lignin, and thus, the content of the polymer increased by 6.2% and 6.4% in the final substrate for *F. velutipes* and *A. aegerita*, respectively (Table S1, Supplementary Material).

*F. velutipes* and *A. aegerita* both proved to be predominantly cellulolytic exotic mushrooms. Cellulolytic fungi can produce a battery of special enzymes, namely cellulases. Cellulases, according to their type of enzymatic activity, can act as endoglucanases, cellobiohydrolases, exoglucanases, or beta-glucosidases [40,44]. They are diverse and consist of a wide range of functional proteins capable of hydrolyzing highly crystalline cellulose [45]. The biodegradation of cellulose and hemicelluloses is likely to depend on similar enzymes. However, hemicelluloses (e.g., pentoses and hexoses) are more heterogeneous than celluloses and thus require more enzymes for effective degradation [46]. 

Xie et al. [47] studied the biodegradation by *F. velutipes* of a composite substrate consisting of 50% ramie’s stalk, 20% cottonseed hull, 25% wheat bran, 4% corn starch, and 1% CaCO3 and computed a value of 119.7% for biological efficiency. The fungus was highly capable of degrading lignin (12.7–32%), cellulose (14.4–30.2%), and hemicellulose (9.3–25.7%), and the enzymes laccase, peroxidase, cellulase, and hemicellulase catalyzed the process. Laccase and peroxidase both acted more effectively than any other enzyme until fruiting, while the cellulase, hemicellulose, and even ligninolytic enzymatic complex determined the biodegradation after fruiting levelled off when the substrate reached a C/N ratio of about 30:1.

### 3.3. Technical Feasibility of Mushroom-Producing Systems on the Balance of Mass

**Figure 5:**
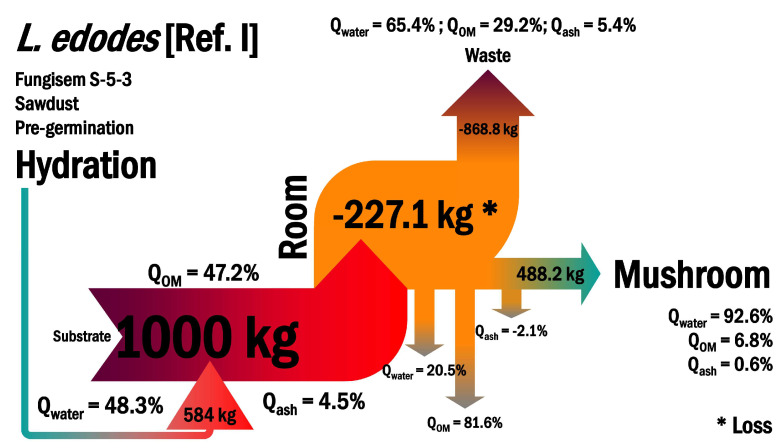
Balance of mass in the system producing *L. edodes* with the support of hydration to a higher level of bioconversion of the substrate into edible mushrooms. * Refers to a mass loss value in the production room.

We designed our systems to be scalable. They consisted of the following key elements: substrate, growing room, fruiting bodies, and waste. We quantified the water, organic matter, and ash at every point to determine the balance of mass and identify which flows could limit production. By computing the mass entering and leaving the physical systems, we could compare them and uncover tractable problems of management. For instance, by introducing 1000 kg substrate (48.3% water, 47.2% organic matter, and 4.5% ash) into the system with an additional level of management by hydration (584 kg), we optimally produced 488.2 kg of *L. edodes*, consisting of 92.6% water, 6.8% organic matter, and 0.6% ash (Figure 5). Although hydration maximized production, thereby resulting in the highest biodegradability and biological efficiency, the room lost an intermediate quantity of mass of 227.1 kg (20.5% water, 81.6% organic matter, and −2.10% ash) and generated 868.8 kg of waste. Such values are important for the final designation of spent mushroom substrates after mushroom production. The residual material from *L. edodes* (Ref. I) could be useful as scaffolds for bioconstruction and green/clean engineering [48].

**Figure 6:**
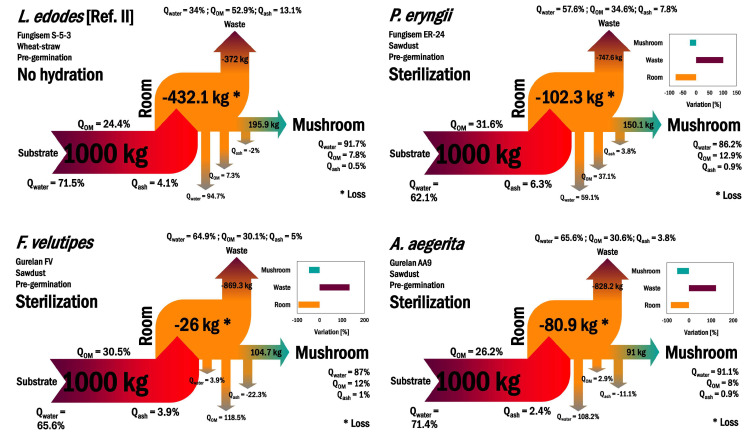
Comparative balance of mass through the systems producing exotic mushrooms. The sublevel bar-plot diagram displays the performance of exotic fungi relative to *L. edodes*. * Refers to a mass loss value in the production room.

The system producing *L. edodes* on a substrate consisting of wheat straw without hydration yielded 195.9 kg of edible mushrooms, making it technically comparable to systems focusing on *P. eryngii* (150.1 kg) (Figure 6); however, less than half this amount was produced in the substrate with sawdust (Ref. I). This difference is due to the absence of mycelial maturation, which in the substrate with sawdust is reached by a longer period of mycelium run (browning process). Certainly, the mycelial layer resulted in less water loss in the cultivation rooms (20.5%) relative to wheat straw (94.7%), although the cereal base generated less waste (372 kg) (Figure 6).

In particular, *P. eryngii*, which had the highest biodegradability and biological efficiency among the tested exotic mushrooms, generated 747.6 kg of spent substrate (57.6% water, 34.6% organic matter, and 7.8% ash), ranking it third in the production of waste. *F. velutipes* generated 869.3 kg of waste (64.9% water, 30.1% organic matter, and 5% ash), ranking first, while *A. aegerita* generated 828.2 kg of waste (65.6% water, 30.6% organic matter, and 3.8% ash), placing it in second rank. The ranking by loss of material straight from the growing room shifted to *P. eryngii* (102.3 kg; 59.1% water, 37.1% organic matter, and 3.8% ash), *A. aegerita* (80.9 kg; 108.2% water, 2.9% organic matter, and −11.1% ash), and *F. velutipes* (26 kg; 3.9% water, 118.5% organic matter, and −22.3% ash). 

Interestingly, the lowest loss of water (3.9%) from *F. velutipes* was even lower than that from *L. edodes* (Ref. I) with mature mycelium (20.5%). This suggests a specific cellular characteristic of the *F. velutipes* mycelium, which could be due to some biochemical compound or morphological structure that reduces/prevents the evaporation of water, since the cultivation time was intermediate (43 days) between *P. eryngii* (38 days) and *A. aegerita* (51 days). Future studies can be developed to elucidate these mechanisms in *F. velutipes*, which demonstrates a high potential for biotechnological application. It would be promising to introduce this/these gene(s) into other edible and medicinal mushroom species to reduce the amount of water applied to the cultivation chambers throughout the harvest cycle.

According to the analysis of the proximal composition of the mushrooms, *A. aegerita* concentrated more water (91.1%) but less organic matter (8%) than the other species. *P. eryngii* proved the least effective at concentrating water (86.7%), which allows a longer shelf life for commercialization. *F. velutipes* had an intermediate water content (87%) and concentrated organic matter (12%) as effectively as *P. eryngii*. We found a similar trend for ash, which ranged from 0.9% for both *P. eryngii* and *A. aegerita* to 1% for *F. velutipes*, although the exotic mushrooms distinctly mineralized the substrate. 

Overall, among the exotic mushrooms, *P. eryngii* produced the largest quantity of fruiting bodies. Additionally, it generated the lowest quantity of waste, potentially making it easier to handle the downstream, whether for recycling or reuse as a base for the formulation of new substrates to enable cheaper, cleaner, and safer production. *F. velutipes* and *A. aegerita* had lower biodegradability. The larger portion of ash (non-combustible) in the waste could make it challenging to reuse waste (SMS) as an alternative feedstock for bioenergy.

## 4. Conclusions

By hydrating through the substrate, we enhanced the growth of *L. edodes* on sawdust; however, it generated a larger quantity of waste after mushroom cultivation. *P. eryngii* could be technically comparable to *L. edodes* without hydration, making it the most reliable option for scaling exotic mushrooms. This fungus demonstrated lignocellulolytic activity by decomposing structural polymers as a whole, similar to *L. edodes*. In contrast, *A. aegerita* and *F. velutipes* could not degrade lignin, indicating that these are both predominantly cellulolytic microorganisms. Analytical insights into biodegradability and how exotic mushrooms and substrates can interact to determine the breakdown of biomolecules are timely and will likely improve decision-making on biomaterials and strains to address particular demands of microbial metabolism. 

## Data Availability

Not applicable.

## Supplementary Materials

The following supporting information can be downloaded at: https://www.mdpi.com/article/10.3390/microorganisms11040897/s1; Table S1: Balance of mass in the systematic cultivation of *L. edodes* and exotic fungi capable of producing edible mushrooms.
