# Supplementary material for: A Comparative Analysis of Biodegradation and Bioconversion of Lentinula edodes and Other Exotic Mushrooms
Source: Microorganisms. 2023 Mar 30;11(4):897. doi: 10.3390/microorganisms11040897 (PMC10142386; doi:10.3390/microorganisms11040897)
Supplement: Supplementary file 1 [file microorganisms-11-00897-s001.zip › microorganisms-2283017-supplementary.pdf]

A Comparative Analysis of Biodegradation and Bioconversion of *Lentinula edodes* and other Exotic Mushrooms

Diego Cunha Zied [1,*], Marcos Antônio da Silva Freitas [1], Bruno Rafael de Almeida Moreira [2], Lucas da Silva Alves [3] and Arturo Pardo-Giménez [4]

[1] Department of Plant Production, College of Agricultural and Technological Sciences, São Paulo State University (Unesp), Dracena 17900-000, SP, Brazil; marcos.freitas@unesp.br
[2] Department of Engineering and Exact Sciences, School of Agricultural and Veterinary Sciences, São Paulo State University (Unesp), Jaboticabal 14884-900, SP, Brazil
[3] Department of Applied Microbiology, School of Agricultural and Veterinary Sciences, São Paulo State University (Unesp), Jaboticabal 14884-900, SP, Brazil
[4] Centro de Investigación, Experimentación y Servicios del Champiñón (CIES), 16220 Quintanar del Rey, Spain
* Correspondence: diego.zied@unesp.br; Tel.: +55-18-3821-8200

Supplementary material

Table S1. Balance of mass into the systematic cultivation of *L. edodes* and exotic fungi capable of producing edible mushroom

Property	*L. edodes* [Ref. I]	*L. edodes* [Ref. II]	*P. eryngii*	*F. velutipes*	*A. aegerita*	*L. edodes* [Ref. I]	*L. edodes* [Ref. II]	*P. eryngii*	*F. velutipes*	*A. aegerita*
	Initial [g element 1000 g^{-1} substrate]					Final [g element 1000 g^{-1} substrate]				
Water	483	715.2	621	656	714	568	126.3	431	563.7	543.5
Dry matter	517	284.8	379	344	286	300.5	245.6	316.5	305.6	284.6
Ash	44.6	41.1	63.1	39	23.6	46.7	48.7	58	43.8	31.7
Organic matter	472.4	243.8	315.9	305	262.4	253.8	196.9	258.5	261.8	252.8
NDF	309.4	177.3	237.6	209.6	228.6	139	137.8	176.4	177.5	207.1
NDS	163	66.4	78.2	95.4	33.8	114.8	59.1	82.1	84.3	45.7
ADF	140.1	126.7	157.2	132.1	164.9	96.7	102.9	126	106.8	145.2
Hemicellulose	169.3	50.7	80.5	77.5	63.7	42.3	34.8	50.3	70.6	61.9
Cellulose	87.4	105.2	106.5	90.2	130.4	78.2	92.3	87.1	62.2	108.5
Lignin	52.7	21.4	50.6	42	34.5	18.5	10.7	38.9	44.6	36.7
Protein	33.4	12.6	22.8	21.5	12.5	19.3	8.5	16.7	18.5	13
Crude fibre	112.2	98.3	134.4	93.2	126.5	76.4	78	105.2	75.5	109
Crude fat	9.8	1.9	4.5	7.7	0.9	8.1	1.5	4.1	7.3	0.8
N-free extract	317.1	131.1	154.1	182.5	122.4	150	108.9	132.4	160.4	130
Total CHOs	429.2	229.4	288.5	275.8	248.9	226.4	186.9	237.6	235.9	239.1
Theoretical CHOs	341.9	124.1	182	185.6	118.6	148.2	94.6	150.6	173.7	130.6

Neutral detergent fiber, NDF; neutral detergent soluble, NDS; acid detergent fiber, ADF; carbohydrates, CHOs.
